# High Neutrophil-to-Lymphocyte Ratio Predicts Cardiovascular Mortality in Chronic Hemodialysis Patients

**DOI:** 10.1155/2017/9327136

**Published:** 2017-02-21

**Authors:** Han Li, Xiangxue Lu, Ruifang Xiong, Shixiang Wang

**Affiliations:** ^1^Department of Blood Purification, Beijing Chao-Yang Hospital, Capital Medical University, Beijing, China; ^2^Nephrology Faculty, Capital Medical University, Beijing, China

## Abstract

The neutrophil-to-lymphocyte ratio (NLR) is a novel simple biomarker of inflammation. It has emerged as a predictor of poor prognosis in cancer and cardiovascular disease in general population. But little was known of its prognostic value in chronic hemodialysis (HD) patients. Here we investigated the association between NLR and cardiovascular risk markers, including increased pulse pressure (PP), left ventricular mass index (LVMI) and intima-media thickness (IMT), and mortality in HD patients. Two hundred and sixty-eight HD patients were enrolled in this study and were followed for 36 months. The primary end point was all-cause mortality and cardiovascular mortality. Multivariable Cox regression was used to calculate the adjusted hazard ratios for NLR on all-cause and cardiovascular survival. We pinpointed that higher NLR in HD patients was a predictor of increased PP, LVMI, and IMT; HD patients with higher NLR had a lower survival at the end of the study; furthermore, high NLR was an independent predictor of all-cause and cardiovascular mortality when adjusted for other risk factors. In conclusion, higher NLR in HD patients was associated with cardiovascular risk factors and mortality.

## 1. Introduction

Cardiovascular disease is the major cause of death in patients with chronic kidney disease, especially in end-stage renal disease (ESRD) patients with chronic hemodialysis (HD). The cardiovascular disease (CVD) mortality in HD patients is much higher than that in general population, and it is not yet entirely explained by traditional risk factors for CVD [1]. Microinflammation is an important factor in the pathogenesis of CVD in HD patients, and it can further accelerate the progression of atherosclerosis [2].

Neutrophil-to-lymphocyte ratio (NLR) is obtained by dividing absolute neutrophil to absolute lymphocyte count. NLR is a novel simple and inexpensive index for assessing inflammation [3]. Emerging evidence suggested that increased NLR was a potential marker of poor prognosis in multiple tumors [4–6] and cardiovascular diseases [7–9] in general population. Cho et al. [10] demonstrated the potential utility of NLR in risk stratification of patients with severe calcific aortic stenosis. Isaac et al. [11] reported that increased NLR was associated with mortality among medical inpatients with multiple chronic conditions. Erturk et al. [12] also demonstrated that an increased NLR was related to higher cardiovascular mortality in patients with peripheral arterial occlusive disease, who were admitted with critical limb ischemia or intermittent claudication. Recently, Ahbap et al. [13] found a significant positive correlation of NLR with hsCRP levels in ESRD patients. In 2012, An et al. [14] reported that NLR was a strong predictor for overall and cardiovascular mortality in peritoneal dialysis patients. Recently, Ouellet et al. [15] reported about NLR as a predictor marker of all-cause survival in incident hemodialysis patients. But to date, little was known of its prognostic value in HD patients. In this present study, we investigated the association between NLR and cardiovascular risk factors, including pulse pressure (PP), left ventricular mass index (LVMI), intima-media thickness (IMT), carotid-femoral pulse wave velocity (cfPWV), and mortality in HD patients.

## 2. Methods

### 2.1. Data Sources

**Figure 1:**
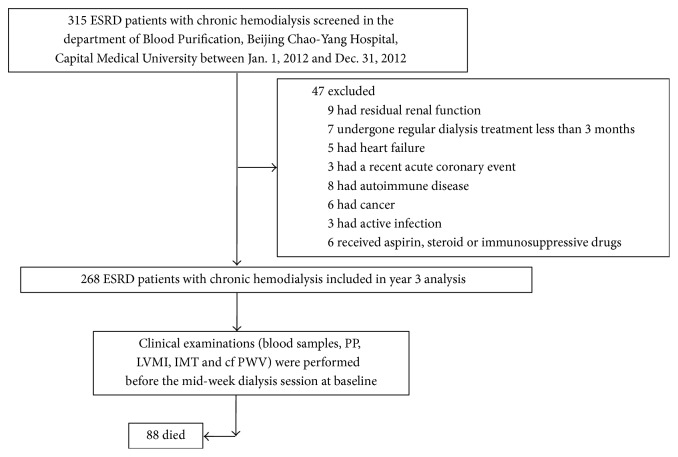
Study flow chart.

A total of 268 ESRD patients on chronic hemodialysis (146 men, 122 women) who were admitted to the department of Blood Purification, Beijing Chao-Yang Hospital, Capital Medical University were recruited from January 1, 2012, to December 31, 2012. The inclusion criteria included ESRD patients having no residual renal function and having undergone regular dialysis treatment for at least 3 months, but without clinical evidence of heart failure, a recent acute coronary event, autoimmune disease, cancer, and active infection and taking aspirin, steroid, or immunosuppressive drugs. A standard questionnaire was adopted from every patient to obtain systematic information regarding conventional cardiovascular risk factors, including hypertension, hyperlipidaemia, diabetes, and family history of cardiovascular disease. All the patients were followed for 36 months. The primary end point was all-cause mortality and cardiovascular mortality. The flow chart of the study was shown as in Figure 1.

The ESRD patients underwent hemodialysis three times a week with standard bicarbonate dialysate (Na^+^ 138 mmol/L, HCO_3_^−^ 35 mmol/L, K^+^ 2.0 mmol/L, Ca^2+^ 1.5 mmol/L, and Mg^2+^ 0.5 mmol/L) and 1.6 m^2^ polysulphone membrane dialysers. Patients were separated into two groups according to common carotid artery plaque, HD patients with and without plaque. The study was performed conform with the declaration of Helsinki and approved by the ethics committee of Beijing Chao-Yang Hospital, Capital Medical University. The written informed consent was obtained from each participant.

### 2.2. Cardiovascular Measurement

Cardiovascular risk markers measurements, including pulse pressure (PP), left ventricular mass index (LVMI), intima-media thickness (IMT), and carotid-femoral pulse wave velocity (cfPWV), were performed before the mid-week dialysis session at baseline.

Blood pressure was measured with a mercury sphygmomanometer after 15 minutes of recumbency. PP was calculated as the systolic blood pressure (SBP) minus the diastolic blood pressure (DBP).

LVMI was evaluated by echocardiography. Left ventricular end diastolic dimension (LVDD), interventricular septum thickness (IVST) and left ventricular posterior wall thickness (LVPWT) were measured. LVMI was calculated and normalized by height^2.7^ (LVMI = LVM/height^2.7^) as previously [16].

IMT was evaluated by common carotid artery ultrasonography as described previously [17]. The mean IMT was calculated as the average of the three readings of bilateral carotid arteries. HD patients with plaque were defined as localized thickening of IMT ≥ 1.2 mm that did not uniformly involve the whole wall of carotid artery.

The common carotid artery stiffness was evaluated by cfPWV. The cfPWV value was measured with the participants in a supine position by using the Complior SP System (Alam Medical, Vincennes, France) [18].

### 2.3. Laboratory Investigations

The fasting blood samples of HD patients were taken from the arterial end of the vascular access immediately before initiation of the mid-week HD session at baseline. The levels of albumin (Alb), alanine transaminase (ALT), aspartate aminotransferase (AST), triglycerides (TG), total cholesterol (Tch), low density lipoprotein-cholesterol (LDL-C), high sensitivity C reactive protein (hsCRP), creatinine (Cr), blood urea nitrogen (BUN), calcium (Ca), and phosphorus (P) were measured by standard laboratory methods using an autoanalyzer. Serum intact parathyroid hormone (iPTH) was determined by immunoradiometric assay.

The blood samples were drawn in plastic vacutainers using EDTA (1 mg/mL of blood) for differential white blood cells count. NLR was calculated as the ratio of neutrophils to lymphocytes from the differential white blood cells count.

### 2.4. Statistical Analysis

All the data were analyzed using statistical software package (SPSS for Windows, Version 20.0, SPSS, USA). Continuous variables data were presented as mean ± standard deviation (±SD). Comparison between groups was performed using independent-samples *t*-test. In addition, spearman correlation was used for univariate analysis and logistic regression was used for multivariate analysis (confidence interval of 95%). Variables entered in multivariate analysis were age, gender, diabetes mellitus, HD duration, LDL-C, hsCRP, PP, LVMI, and IMT (≥1.2 mm, plaque). NLR cut-off value used in survival curves was determined by a receiver operating characteristic (ROC) curve. Survival curves were estimated by Kaplan-Meier analysis and compared by the log rank test. A Cox regression model was used to identify predictors of mortality. A *P* value <0.05 was considered statistically significant.

## 3. Results

### 3.1. Demographic, Clinical, Laboratory, and Vascular Parameters of the Studied Population

A total of 268 HD patients with a mean age of 48.7 ± 10.9 years (range 21–78 years) and a mean dialysis period of 45.9 ± 32.5 months (range 4–146 months) were enrolled in this study. The baseline demographic, clinical, biochemical, and vascular characteristics of patients were described as shown in Table 1.

### 3.2. Characteristics of HD Patients with and without Common Carotid Artery Plaque

According to the localized thickness of IMT, we found that about 44.4% HD patients had plaque in common carotid artery. Mean level of NLR in all HD patients was 3.36, but the HD patients with plaque had higher level of NLR (*n* = 119). There were no significant differences with respect to the following variables between both groups: age, sex distribution, dialysis duration, diabetes, smoking, KT/V, Hb, serum creatinine, BUN, TG, Tch, and LDL-C in HD patients with plaque or without plaque. But interestingly, HD patients with plaque also had higher serum hsCRP level (Table 2).

### 3.3. Correlation of NLR with Cardiovascular Risk Factors in HD Patients

**Figure 2:**
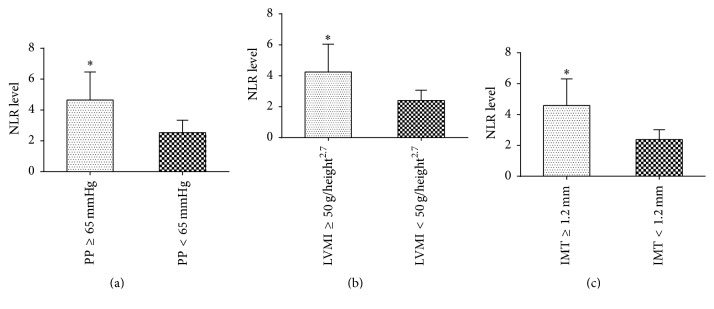
NLR level in HD patients with CVD risk factors. (a) HD patients with higher PP (≥65 mmHg) had higher NLR level. *∗* indicates a significant difference between the PP (≥65 mmHg) group and PP (<65 mmHg) group (*P* < 0.01); (b) HD patients with higher LVMI (≥50 g/height^2.7^) had higher NLR level. *∗* indicates a significant difference between the LVMI (≥50 g/height^2.7^) group and LVMI (<50 g/height^2.7^) group (*P* < 0.01); (c) HD patients with higher IMT (≥1.2 mm) had higher NLR level. *∗* indicates a significant difference between the IMT (≥1.2 mm) group and IMT (<1.2 mm) group (*P* < 0.01).

As shown in Figures 2(a), 2(b), and 2(c), HD patients with higher PP (≥65 mmHg), LVMI (≥50 g/height^2.7^), and IMT (≥1.2 mm) had significantly higher NLR levels (all *P* < 0.01).

By testing using univariate analysis, NLR levels were positively correlated with LVMI (*r* = 0.566; *P* < 0.01), PP (*r* = 0.579; *P* < 0.01), cfPWV (*r* = 0.935; *P* < 0.01), IMT (*r* = 0.578; *P* < 0.01), plaque (*r* = 0.776; *P* < 0.01), and hsCRP (*r* = 0.552; *P* < 0.01). There was no correlation with age, gender, dialysis duration, smoking, diabetes, and serum LDL, as shown in Table 3.

Furthermore in multivariate analysis, NLR was an independent predictor of cardiovascular risk markers, high PP (PP ≥ 65 mmHg, OR = 3.056, 95% CI: 2.051–4.553, *P* < 0.01), high LVMI (LVMI ≥ 50 g/height^2.7^, OR = 3.457, 95% CI: 2.271–5.264, *P* < 0.01), and plaque (IMT ≥ 1.2 mm, OR = 5.248, 95% CI: 3.178–8.667, *P* < 0.01).

### 3.4. NLR Level in HD Patients with Cardiovascular Death and All-Cause Death

**Figure 3:**
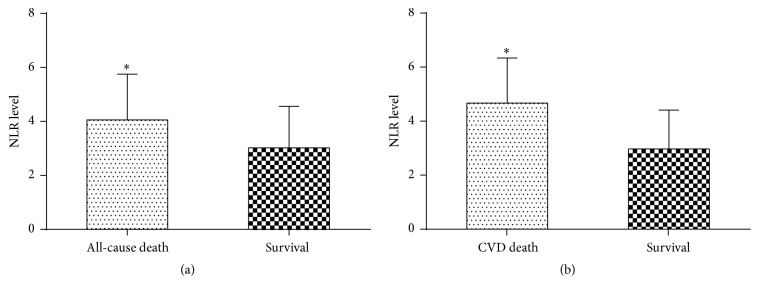
NLR level in HD patients with CVD and overall death. (a) HD Patients who died from overall causes had higher NLR level. *∗* indicates a significant difference between the all-cause death group and survival group (*P* < 0.01); (b) HD Patients who died from cardiovascular causes had significantly higher NLR level. *∗* indicates a significant difference between the CVD death group and survival group (*P* < 0.01).

In this study, 88 of 268 (32.8%) patients died from overall causes during the 36-month period, and 62 of 88 (70.5%) patients died from cardiovascular causes. HD patients with cardiovascular death had higher level of NLR (CVD death versus survival, 4.67 ± 1.66 versus 2.96 ± 1.43; *P* < 0.01). And HD patients who died from overall causes had higher NLR level (4.06 ± 1.69 versus 3.02 ± 1.53; *P* < 0.01) (Figures 3(a) and 3(b)).

### 3.5. NLR More than or Equal to 3.5 Was Associated with High All-Cause and Cardiovascular Death in HD Patients

**Figure 4:**
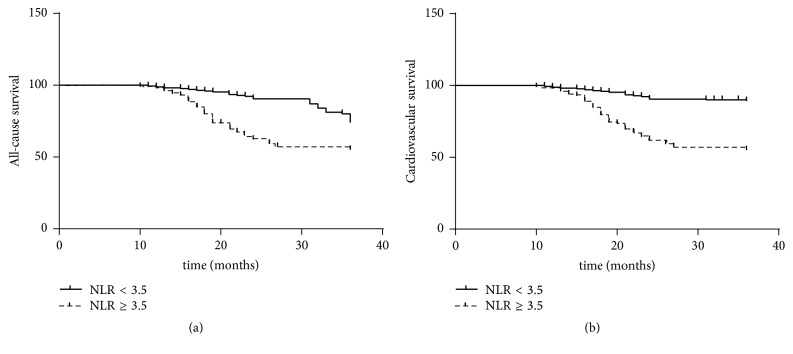
NLR ≥ 3.5 was associated with higher all-cause and cardiovascular death. (a) NLR ≥ 3.5 had a significant higher 36-month all-cause mortality in HD patients (log rank = 15.28; *P* < 0.01); (b) NLR ≥ 3.5 had a significant higher 36-month cardiovascular mortality in HD patients (log rank = 43.54; *P* < 0.01).

The cut-off value of NLR determined by ROC curve analysis was 3.5 (AUC: 0.847; 95% CI: 0.801–0.892; 98.4% sensitivity; 79.1% specificity). Kaplan-Meier analysis showed that overall causes (log rank = 15.28; *P* < 0.01) and cardiovascular diseases (log rank = 43.54; *P* < 0.01) were responsible for a significant lower 36-month survival in HD patients with mean NLR level more than and equal to 3.5 (Figures 4(a) and 4(b)).

Cox regression analysis showed that NLR was a significant predictor of all-cause mortality (HR = 1.695; 95% CI = 1.288–2.231; *P* < 0.01) and cardiovascular mortality (HR = 1.379; 95% CI = 1.162–1.637; *P* < 0.01) in HD patients, using models adjusted for demographic and clinical covariates, which were age, gender, diabetes mellitus, HD duration, LDL-C, hsCRP, PP, LVMI, and plaque (IMT ≥ 1.2 mm).

## 4. Discussion

In this study, we evaluated the prognostic value of NLR for cardiovascular risk factors and mortality in HD patients. The results indicated that NLR was an independent predictor of higher PP, LVMI, and IMT. Interestingly, we further found that NLR more than or equal to 3.5 was a predictor of all-cause mortality and cardiovascular mortality in HD patients.

Previous studies have illustrated the predictive value of NLR as a novel inflammation marker in patients with cardiovascular diseases in general population. In the hypertension patients, the NLR value increased and positively correlated with hyperhomocysteinemia [19]. In the pathogenesis of aneurysm of the ascending aorta in hypertensive patients, NLR as a marker of inflammation may play an important role [20]. In patients with symptomatic intermediate carotid artery stenosis, NLR was increased and the increased NLR value was an independent variable for carotid artery plaques to become symptomatic [21]. In ischemic stroke patients, dynamic change of NLR has been shown to predict hemorrhagic transformation after thrombolysis [22]. In patients with ST-segment elevation myocardial infarction, NLR was related to electrocardiographic sign of spontaneous reperfusion [23]. In patients undergoing nonurgent percutaneous coronary intervention, a higher NLR increased the risk of periprocedural myocardial infarction [24]. NLR was also significantly associated with microvascular disease in asymptomatic subjects [25]. Furthermore, it has been reported recently that elevated NLR was associated with worse overall survival in noncancer patients [26]. In patients with peripheral arterial occlusive disease, an increased NLR was related to higher mortality [27]. In patients with advanced heart failure, elevated NLR was associated with increased mortality or heart transplantation risk [28]. Meanwhile, Durmus et al. [29] found that NLR was higher in heart failure patients and a cut-off value of 5.1 for NLR can predict death in heart failure patients.

However, the association between NLR and cardiovascular disease has been little investigated in CKD patients. Tatar et al. [30] found the basal NLR was an independent predictor of death in geriatric patients with stage 3–5 chronic kidney disease. Kocyigit et al. [31] demonstrated that patients with a high NLR had worse prognosis and significantly faster progression to the dialysis compared with those with a low NLR. Solak et al. [32] reported that NLR was independently related to endothelial dysfunction and could predict composite cardiovascular endpoints independent of traditional confounding factors in patients with moderate to severe CKD. But thus far, little was known of the prognostic value of NLR in hemodialysis patients. In this present study, we investigated the association between NLR and cardiovascular risk factors and mortality in HD patients, and we believe that the current study will provide us new enlightenment and direction in this area.

Chronic inflammation is prevalent in patients with chronic kidney disease and may contribute to morbidity and mortality among dialysis patients [33]. Increased inflammation in ESRD contributes to cardiovascular morbidity, a leading cause of mortality in these patients. Biomarkers have played a significant role in the prediction, diagnosis, and treatment of cardiovascular disease outcomes including myocardial infarction, congestive heart failure, and stroke [34]. The role of inflammatory markers in cardiovascular diseases has been studied extensively and a consistent relationship between C reactive protein and cardiovascular diseases has been established in the past [35]. NLR, a novel biomarker for assessing inflammation, has been getting widely used to identity patients with various illness. NLR is a biomarker that integrates two WBC subtypes representing two inversely and related immune pathways. It was easily calculated from differential WBC counts, more stable for measurement than the individual WBC counts, and less affected by conditions that could change the individual cell counts [36]. The recent remarkable observation has been that NLR has a greater predictability than total WBC count or neutrophil count as a marker in cardiovascular diseases and was slowly emerging as an independent useful prognostic parameter in cardiovascular diseases [37]. According to our present study, an easy and inexpensive laboratory measure of NLR might provide significant information regarding cardiovascular risk factors and mortality in HD patients.

Neutrophil extracellular traps (NETs), first discovered in 2004 by Brinkmann et al. [38], are formed and released by activated neutrophils during the process of NETosis in which the nuclear material is released into extracellular space, including DNA, citrullinated histones, and enzymes of neutrophil granule [39]. This discovery casts a new light on the role of neutrophils in the nonspecific immune response of the body. Although the beneficial effect of NETs in the fight against pathogens has been confirmed in many clinical findings, further evidence has been provided that NETs may promote inflammatory reactions and cause damage to tissues [40]. Additionally, circulating cell-free DNA, a maker of NETs formation, has been demonstrated to promote inflammation and to predict mortality in HD patients [41, 42]. Meanwhile, Qin et al. reported that NETosis markers, including neutrophil elastase and proteinase 3, were positively correlated with absolute neutrophil count in type 1 diabetes patients [43]. All of these findings suggest that the formation of NETs may be one of the possible mechanisms by which an increased NLR is related to higher mortality. Unfortunately, there is little research on the relationship between NETs and NLR in HD patients.

Some limitations of this study should be acknowledged. First, in our study, the patients were selected only from the dialysis center in our hospital instead of from a general population; thus this may not be an accurate reflection of the general population. Second, we measured NLR for only one time while serial measurements would have been more informative. Third, we only investigated the effect of the NLR value on the cardiovascular risk factors and mortality in HD patients. But we have not yet compared the predictive role of NLR with other simple inflammatory markers, such as total white blood count and platelet to lymphocyte ratio; thus we did not draw a conclusion which was the best biomarker to predict the cardiovascular risk factors and mortality in HD patients. Meanwhile, although we found that an increased NLR was related to higher mortality in HD patients, its possible molecular mechanism was still not clear.

## 5. Conclusions

This study demonstrated that a high NLR value was associated with the increased risk of cardiovascular disease. NLR more than or equal to 3.5 predicted all-cause and cardiovascular death in HD patients. Thus, NLR, which is easy to access and inexpensive, may be a novel biomarker for assessing inflammation and identifying high risk for cardiovascular disease and death in HD patients. However, there are still many problems needing further research, such as the mechanism of the effect of high NLR value on the cardiovascular disease and death in HD patients and the effect of high NLR on the specific kind of cardiovascular diseases, so that we will finally find a cheap, reliable, and independent prognostic biomarker of cardiovascular disease and death in HD patients.

## Figures and Tables

**Table 1:** Demographic and biochemical parameters of the studied population.

Items	Patients (*n* = 268)
Age, years	48.7 ± 10.9
Gender, male, *n* (%)	149 (55.6%)
Primary disease	
Chronic glomerulonephritis, *n* (%)	106 (37.1%)
Hypertensive nephropathy, *n* (%)	31 (11.6%)
Diabetic nephropathy, *n* (%)	45 (16.8%)
Chronic interstitial nephritis, *n* (%)	22 (8.2%)
Polycystic kidney disease, *n* (%)	15 (5.6%)
Unknown, *n* (%)	49 (18.3%)
Dialysis duration, months	45.9 ± 32.5
Smoking, *n* (%)	71 (26.5%)
Diabetes, *n* (%)	49 (18.3%)
NLR	3.36 ± 1.65
LVMI, g/height^2.7^	53.3 ± 10.5
LVMI > 50 g/height^2.7^, *n* (%)	140 (50.2%)
PP, mmHg	67.6 ± 18.1
PP > 65 mmHg, *n* (%)	105 (39.2%)
cfPWV (mm/s)	14.7 ± 5.9
IMT (mm)	1.15 ± 0.16
Plaque, *n* (%)	119 (44.4%)
KT/V	1.36 ± 0.03
Hb, g/L	114.9 ± 12.7
Alb, g/L	36.3 ± 3.5
ALT, U/L	14.9 ± 5.8
AST, U/L	14.3 ± 6.2
TG, mmol/L	1.82 ± 1.17
Tch, mmol/L	4.24 ± 0.86
LDL-C, mmol/L	2.24 ± 0.63
hsCRP, mmol/L	4.85 ± 3.40
Cr, mol/L	913.6 ± 167.9
BUN, mmol/L	24.8 ± 6.0
Ca, mmol/L	2.29 ± 0.33
P, mmol/L	1.93 ± 0.60
iPTH, pg/ml	270.6 ± 125.8
RASI, *n* (%)	203 (75.7%)
CCB, *n* (%)	209 (78.0%)
*β*-Blocker, *n* (%)	79 (29.1%)

Values are means ± SD, unless specified otherwise.

NLR: neutrophil to lymphocyte ratio; LVMI: left ventricular mass index; PP: pulse pressure; cfPWV: carotid–femoral pulse wave velocity; IMT: intima-media thickness; Hb: hemoglobin; Alb: albumin; ALT: alanine transaminase; AST: aspartate aminotransferase; TG: triglyceride; Tch: total cholesterol; LDL-C: low density lipoprotein-cholesterol; hsCRP: high sensitivity C reactive protein; Cr: creatinine; BUN: blood urea nitrogen; Ca: calcium; P: phosphorus; iPTH: intact parathyroid hormone; RASI: renin angiotensin system inhibitor; CCB: calcium channel blocker; *β*-blocker: *β*-receptor blocker.

**Table 2:** Characteristics of HD patients with and without plaque.

Items	HD/nonplaque group (*n* = 149)	HD/plaque group (*n* = 119)	*t*/*χ*^2^ value	*P* value
Age, years	48.3 ± 10.9	49.3 ± 10.9	0.773	0.440
Gender, male/female	32/28	30/23	0.082	0.774
Dialysis duration, months	44.5 ± 30.8	47.7 ± 34.6	0.787	0.432
Smoking, no. (%)	16 (26.7)	14 (26.4)	0.181	0.671
Diabetes, no. (%)	11 (18.3)	10 (18.9)	1.064	0.302
NLR	2.38 ± 0.63	4.59 ± 1.72^a^	14.484	0.000
KT/V	1.36 ± 0.28	1.36 ± 0.29	0.293	0.770
Hb, g/L	115.5 ± 12.9	114.2 ± 12.5	0.840	0.402
Alb, g/L	36.4 ± 3.6	36.2 ± 3.4	0.438	0.662
ALT, U/L	15.2 ± 6.0	14.6 ± 5.5	0.779	0.437
AST, U/L	14.6 ± 6.8	13.9 ± 5.5	0.940	0.348
TG, mmol/L	1.89 ± 1.26	1.72 ± 1.05	1.141	0.255
Tch, mmol/L	4.31 ± 0.95	4.15 ± 0.74	1.561	0.120
LDL-C, mmol/L	2.26 ± 0.65	2.22 ± 0.61	0.466	0.642
hsCRP, mmol/L	3.06 ± 1.88	7.09 ± 3.53^a^	11.958	0.000
Cr, mol/L	917.0 ± 167.6	909.3 ± 168.9	0.372	0.710
BUN, mmol/L	25.0 ± 5.8	24.6 ± 6.1	0.588	0.557
Ca, mmol/L	2.28 ± 0.31	2.30 ± 0.35	0.513	0.608
P, mmol/L	1.96 ± 0.59	1.89 ± 0.62	1.023	0.307
iPTH, pg/ml	274.6 ± 126.4	265.4 ± 125.3	0.590	0.556
RASI, no. (%)	43 (71.7)	42 (79.2)	0.285	0.593
CCB, no. (%)	45 (75.0)	43 (81.1)	0.691	0.406
*β*-Blocker, no. (%)	17 (28.3)	17 (32.1)	2.325	0.127

Values are means ± SD, unless specified otherwise.

^a^ *P* < 0.01, compared with HD/nonplaque group.

NLR: neutrophil to lymphocyte ratio; Hb: hemoglobin; Alb: albumin; ALT: alanine transaminase; AST: aspartate aminotransferase; TG: triglyceride; Tch: total cholesterol; LDL-C: low density lipoprotein-cholesterol; hsCRP: high sensitivity C reactive protein; Cr: creatinine; BUN: blood urea nitrogen; Ca: calcium; P: phosphorus; iPTH: intact parathyroid hormone; RASI: renin angiotensin system inhibitor; CCB: calcium channel blocker; *β*-blocker: *β*-receptor blocker.

**Table 3:** Correlation coefficients for NLR and other variables in HD patients.

Variables	*R*	*P *value
Age	0.005	0.931
Gender	0.008	0.899
Smoking	0.006	0.919
Diabetes	0.042	0.489
Dialysis durations	0.055	0.369
LDL-C	0.002	0.978
hsCRP	0.552	0.000
LVMI	0.566	0.000
PP	0.579	0.000
IMT	0.578	0.000
Plaque	0.776	0.000
cfPWV	0.935	0.000

LDL-C: low density lipoprotein-cholesterol; hsCRP: high sensitivity C reactive protein; LVMI: left ventricular mass index; PP: pulse pressure; IMT: intima-media thickness; cfPWV: carotid–femoral pulse wave velocity.
